# How do high-level African directors of policy and planning operationalize health equity? Findings from a regional survey

**DOI:** 10.1371/journal.pgph.0004384

**Published:** 2025-03-26

**Authors:** Michelle Amri, Anna Socha, Elisabeth Huang, Daniel Steel, Danielle Jacobson, Jesse B. Bump

**Affiliations:** 1 The W. Maurice Young Centre for Applied Ethics, School of Population and Public Health, University of British Columbia, Vancouver, British Columbia, Canada; 2 Independent Researcher, Basel, Basel-Stadt, Switzerland,; 3 Independent Researcher, Toronto, Ontario, Canada,; 4 Institute for Better Health, Trillium Health Partners, Mississauga, Ontario, Canada; 5 Takemi Program in International Health, Harvard T.H. Chan School of Public Health, Harvard University, Boston, Massachusetts, United States of America; 6 Bergen Centre for Ethics and Priority Setting, University of Bergen, Bergen, Norway; UNC Gillings School of Global Public Health: The University of North Carolina at Chapel Hill Gillings School of Global Public Health, UNITED STATES OF AMERICA

## Abstract

This study investigates the operationalization of health equity and associated challenges faced by directors of policy and planning and those in related positions in the African Region. The results of this study demonstrate that health equity is generally a priority for policymakers in practice and is operationalized in four ways: (i) targeting priority groups, (ii) focusing on the entire population; (iii) through procedural justice; and (iv) operationalizing population health data. The targeted approach, which predominated, tended to focus on individuals with lower socioeconomic status, maternal and child health services, and select infectious diseases (e.g., HIV/AIDS). The population-wide approach entailed the inclusion of health equity in institutional laws, constitutions, and national policies with efforts to ensure access to health services for all. Procedural justice largely focused on the inclusion of stakeholders in decision-making processes. Lastly, operationalizing population health data was noted to guide policy and planning to address health inequities, often through aiding in the selection of priority groups or areas for intervention and monitoring and evaluation actions focused on improving health equity. Four main domains of challenges for incorporating health equity emerged relating to: (i) understanding health equity, (ii) governance, (iii) resources, and (iv) lack of data. Our recommendations are two-fold: (i) we recommend that researchers focus on improving understandings of health equity among policymakers through knowledge translation and exchange, and (ii) we recommend that policymakers and those working within donor organizations focus on reforming any top-down processes through which priorities are set and decisions are made.

## Author summary

Health equity remains a key goal for policymakers globally. Our study explores how health equity is understood and put into practice by directors of policy and planning and those in related positions in the African Region. After distributing a survey, we found that health equity is prioritized in four ways: (i) by targeting specific groups, (ii) by focusing on the entire population, (iii) through direct inclusion of the population in decision-making processes, and (iv) by using health data for policy and planning decisions. Notably, the most common approach focused on targeted policies and interventions for individuals with lower socioeconomic status, maternal and child health services, and select infectious diseases. Despite these actions on health equity, policymakers shared that challenges remain relating to: (i) understanding health equity, (ii) governance, (iii) resource constraints, and (iv) lack of data. We recommend that: (i) researchers focus on improving understandings of health equity among policymakers through sharing knowledge, and (ii) policymakers and those working within donor organizations focus on reforming any processes where decisions and priorities are imposed, non-participatory, or not inclusive (i.e., top-down processes).

## Background

Health equity has gained prominence in global and public health over the last several decades. For example, equity has been globally recognized in the Universal Declaration of Human Rights adopted in 1948 [1] and in the push for “health for all by the year 2000” by the World Health Organization (WHO) beginning in 1978 [2]. Although there is no universally agreed upon definition of health equity, the characterisation of inequity in health commissioned by the WHO refers to “differences which are unnecessary and avoidable but, in addition, are also considered unfair and unjust” [3]. Applying this definition requires a judgement to determine what is in fact unfair [3]. Despite the WHO arguably setting the course for heightened attention to and discussion on health equity in global and public health, this concept has drawn scrutiny showing that the WHO has applied inconsistent conceptualizations of health equity [4–9].

Given the multifarious conceptualizations of health equity and their implications for implementation, understanding the actions taken by directors of policy and planning becomes particularly important. Directors and those in related positions of policymaking hold power to influence policy design and implementation, which entails determining which populations and sub-groups are included, or even considered, in policymaking. This is particularly helpful in the “declining world” where beginning such a dialogue on fair resource distribution and political discourse more generally is needed [1]. As such, this study expands on previous work, which found that respondents understood health equity in various ways, whereby the concept of health equity aligned with different theories of distributive justice [10], and seeks to determine how high-level policymakers in the African Region approach health equity through policy and practice. In other words, what are the foci of efforts and what challenges persist to further operationalizing health equity?

## Methods

### Ethics statement

The WHO’s Regional Office for Africa endorsed the study and Harvard University’s institutional research board provided an ethics exemption for this study (protocol number: IRB21-1176). The recruitment period was from March 31, 2022 to September 1, 2022. Consent was a condition of completing the written survey. The survey contained an introductory statement on the instrument itself explaining the study, stressing the completely voluntary option to complete it, and specifying that participants can decline to participate in any part of this study for any reason and can end their participation at any time. More details on the survey content are noted below.

### Study design

An 18-item survey was developed and administered to directors of policy and planning and those in related positions to glean how health equity is operationalized. A survey was chosen to both provide us with a broad understanding and collect data from as many respondents as possible, given the busy nature of policymakers’ jobs, maximizing the number of individuals we could reach. M.A.’s previous research on the concept of health equity as operationalized by the WHO informed the development of survey questions. Once an initial draft of the survey was complete, J.B.B., a global and public health scholar who is well-versed in qualitative methods, reviewed all survey items.

Individual consultations were then undertaken by M.A. with six directors within national governments across the African Region. These consultations focused on sharing the primary objectives of the study, the value that this study seeks to add, and the survey questions. Feedback was collated and survey items were accordingly revised. For instance, it was suggested to indicate that directors can also include managers and other high-level policymakers to recognize differing job titles across countries and collect data on the specifics of individuals’ roles.

### Distribution strategy

An invitation to complete a Qualtrics survey was disseminated by email which included a description of the study’s purpose, eligibility to participate, what participation entails, and benefits and risks to participation. The email distribution list included 481 email addresses from those who were invited to the WHO’s Fifth Health Sector Directors’ Policy and Planning Meeting for the WHO African Region [11]. The email invitation was also shared with 55 non-duplicate email addresses that were taken from an online list of Ministry of Health policy focal points.

It was not possible to discern if all email addresses on the complete distribution list were relevant to the study. For instance, translators and other similar individuals were included on the email distribution list from the WHO’s Fifth Health Sector Directors’ Policy and Planning Meeting. Additionally, some email addresses had university domains rather than government domains. However, it is possible that the owner of an email address with a university domain could have been an alumnus who became employed as a civil servant. Therefore, the entire email list was included in the survey distribution and email addresses were not excluded based on having a university-affiliated domain. Email addresses with any of the following institutional domains were excluded from the distribution list to reach policymakers as opposed to global actors: who.int; unicef.org; giz.de; unfpa.org; pasteur.sn; kemri-wellcome.org; gavi.org; gatesfoundation.org; dfid.gov.uk; and worldbank.org. Duplicate emails were excluded from the distribution list.

Eligibility to participate was further determined via a sociodemographic screening at the beginning of the survey. The sociodemographic screening gathered information about where participants work, the focus of their work, and the government level at which they work. Participants were eligible to participate if they (i) were a director of policy and/or planning or similar role, and (ii) worked in any government in the WHO Region of Africa.

### Pilot testing of the survey

From the complete email distribution list, 50 email addresses were randomly selected to pilot the survey to determine if any changes to survey questions were needed before distributing the invitation to participate to the remaining email addresses. Pilot respondents’ responses appropriately answered the survey questions indicating that the questions posed were clearly interpreted. Therefore, no further changes to the survey were required.

### Response rate

The invitation to participate was distributed to a total of 536 email addresses. There were 282 emails that bounced back and 254 emails that did not bounce back. Therefore, a maximum of 254 potential respondents received the email invitation. The actual number received would be lower in ways we cannot estimate, including how many were diverted by spam filters or how many went to inactive accounts. All potential respondents were sent a total of four emails at approximately one-week intervals with the invitation to participate in the survey. Subsequently, a WHO official sent one follow-up email to encourage participation in the study.

There were 42 respondents (although one respondent did not complete the survey in full) across 18 of the 47 countries within the African Region (as outlined by the WHO). The response rate was 16.5% based on the 254 potential respondent figure. Given that the list of potential respondents who received the email invitation to participate in this survey included non-directors (e.g., translators), it is important to note that 16.5% is not an accurate representation of the response rate of *eligible* participants. The email invitation included information on eligibility. There were no ineligible respondents who attempted to complete the survey. Had a non-eligible respondent attempted to complete the survey, they would have been excluded by the sociodemographic screening.

### Survey content

The survey contained an explanatory preamble, noted that participation is voluntary, outlined the expected time commitment of participation (5-15 minutes), and pointed out the potential associated benefits (e.g., inform policy work that aims to improve global population health) and risks (e.g., a very small potential risk of being identified through participation). Great care was taken to protect participants’ anonymity, for example, by excluding country-specific details in this article. The preamble also clearly indicated that the respondents could decline to participate without consequence. At the end of the survey, respondents were invited to participate in a follow-up interview and were thanked for their time and participation. These interviews have yet to be conducted as they are planned for a subsequent study.

The survey collected demographic information and asked a series of questions focused on health equity in policy and practice. The questions for which responses were analyzed in this article related to the consideration of health equity in participants’ government departments, how participants directly incorporate health equity into their practice, particular policies or programs that address health equity, and challenges to incorporating health equity in policy and planning work.

### Data analysis

Survey responses were collected in English, French, and Portuguese and were subsequently translated using DeepL software [12]. There was no set coding framework or *a priori* codes. E.H. and A.S. independently inductively coded the English responses and responses translated to English, guided by Braun and Clarke’s steps for thematic analysis [13–15]. E.H. used NVivo12 [16] and A.S. manually coded the survey responses in Microsoft Word. Once the coding was complete, E.H., A.S., and M.A. met to discuss all codes, noting convergences and divergences, and the relevant emergent themes. There was a majority of overlap in codes and their underlying meaning and divergences were few and easily agreed upon; when one author had a code that the other missed, consensus was consistently reached for its inclusion. Three overarching themes, which were not mutually exclusive, emerged from the data related to how respondents acted on health equity. It is important to note that these themes emerged from the data through the lens of the authors’ analysis and were not explicit approaches described by survey respondents. The themes, and subsequent sub-themes, are detailed in the results below.

## Results

The results of this study demonstrate that policymakers expressed that they generally sought health equity in practice. Approximately 90% of respondents (n = 37/41, where the denominator reflects the number of responses per question) agreed that health inequity is considered or actions are implemented for health equity in their government departments. Of the respondents who agreed that health inequity is considered in their government departments, all unanimously answered “yes” when asked “Do you think equity should be further considered or embedded in Ministry of Health work?” (n = 37/37; 100%). Evidently, respondents unanimously felt there is more work to be done around health equity.

In terms of actions taken, high-level directors of policy and planning considered health equity in policy and practice by: (i) targeting priority groups, (ii) focusing on the entire population, (iii) through procedural justice, and (iv) operationalizing population health data toward health equity. Further, key challenges to incorporating health equity in policy and practice were uncovered and centred on governance, resources, external factors, and lack of data. These are detailed below.

### How respondents sought health equity

Two main approaches for addressing health equity emerged. Most respondents mentioned the prioritization of a particular population or disease groups, thus taking a targeted approach. Some respondents alluded to their government’s policies and/or practices as ensuring services are available to all, thus taking a population-wide approach. Participants also discussed procedural justice and operationalizing population health data in seeking health equity. Although not mutually exclusive, more respondents referred to the first approach as their overall approach. These approaches in which health equity is considered in policy and practice are described below.

#### 1. Targeting priority groups.

In discussing how health equity was operationalized, most responses aligned with prioritizing target populations. Respondents prioritized the needs of “vulnerable” or “marginalized” populations who are at a “disadvantage” — without specification around the disadvantage — particularly when designing plans. Most discussions of taking a targeted approach aligned with (i) targeting those with a lower socioeconomic status, (ii) targeting maternal and child health, and (iii) targeting groups with specific health conditions, especially human immunodeficiency virus/acquired immunodeficiency syndrome (HIV/AIDS). Each of these three are described below.

##### (i) Targeting lower socioeconomic status:

Respondents commonly expressed targeting those with a lower socioeconomic status by removing financial barriers at the service delivery level through “[e]stablish[ing] free and subsidized health care programs.” These programs “facilitate[d] access to health care services for “vulnerable groups”. For example, one respondent described subsidized programs targeting specific disease services such as “Free health care program for people living with HIV, subsidy of caesarean sections by the State (90% of the costs), program of care for pregnant women, extended vaccination program…”. There were also references made regarding free services to particular populations such as “exemption from user fees for populations in rural areas that live in the catchment areas of mission health providers and there is no Government facility nearby”. Other respondents explained how health care more generally “is free at the point of access”. This sentiment, and the language around it, was echoed by multiple respondents, with one emphasizing how this “free health care at the point of access…serves as ‘the main equity policy’”.

In addition to offering free health services to address health inequity, other financial-related initiatives to promote equity included: health benefits packages; health equity funds (e.g., financial support for the poor at the health facility level); resource tracking for future health investment; and social insurance. There were some references made to the concept of Universal Health Coverage (UHC) (defined as “ensuring that everyone, everywhere can access the full range of health services they need without financial hardship” [17]) and health insurance. We classified UHC within this theme of targeting those with a lower socioeconomic status due to respondents’ common application of the term alongside financial initiatives for the “poor”. For example, a respondent mentioned a “universal health insurance scheme” and another mentioned their National Health Insurance (NHI) policy: “[the NHI] is a health financing policy that seeks to create a single health system for all [citizens]. With the NHI, it is hoped that resources will be shared equally irrespective of ability to pay.”

##### (ii) Targeting maternal and child health:

Respondents described a particular focus on aspects of gender within policy and planning for health equity. Some respondents expressed prioritizing healthcare delivery for women and children, and that their “research projects are focused on making evidence-based high-impact interventions available to disadvantaged and hard-to-reach women and children.” Other respondents provided specific examples illustrating the strong focus on maternal and child health in both policies and programs. For example, one respondent stated that there are policies that favour pregnant women and children, while another mentioned a policy of free care for pregnant women and children. Initiatives described included a program that offers care to pregnant mothers (this program was national in scope), maternal and child health programs for family planning, and expanded maternal and child health programs. Two respondents also mentioned caesarean section subsidy programs, including one that specified that the State covers 90% of the cost. Another respondent shared that one measure within the “array of policies” and “programmatic interventions to ensure health equity” was “the creation of a Gender Focal Desk at the Ministry of Health”. This Gender Focal Desk “ensure[d] that … the interest of women and female[s] [we]re carefully considered in health policies and actions.” Other respondents noted how gender can become entangled with other important health issues: “[in] the framework of our health program, the integration of equity translates into taking into account the notion of gender which has an impact on the HIV epidemic”.

Interestingly, one respondent noted the lack of consideration of children by stating: “In the framework of our health program, the integration of equity translates into taking into account the notion of gender which has an impact on the HIV epidemic. But also on the age, indeed the first treatments and interventions of [the] fight against AIDS were in favor of the adults leaving for account the children”. We understand this quote to mean that interventions taken to address AIDS are focused on adults, leaving children overlooked in their respective contexts.

##### (iii) Targeting groups by specific health conditions, especially HIV/AIDS:

Most references to specific conditions were related to HIV/AIDS. One respondent noted policies “in favor of people vulnerable to various diseases (HIV,...).” Despite some noted specificity regarding one particular program that tackles AIDS (i.e., “PNLS [national program to fight AIDS]),” most references were general, referring broadly to “the HIV program,” “free health care program for people living with HIV,” or programs financed by donors related to HIV/AIDS amongst other diseases such as malaria, tuberculosis, and neglected tropical diseases.

There were other conditions mentioned less frequently than HIV/AIDS. For example, there were general references to a malaria program (e.g., “care for simple and severe malaria”), “free care for tuberculosis patients,” and an “albendazole supplementation program,” which can be helpful for intestinal parasite infections. There was also the mention of a more specific national program to address neglected tropical diseases. Of note was that “non-communicable diseases (diabetes, hypertension, etc.) do not interest donors.”

#### 2. Focusing on the entire population.

Respondents indicated that health equity is sought by focusing on the entire population. This population-wide approach was reflected in multiple mediums including: (i) institutional laws, constitutions, and national policies and (ii) ensuring access to health services for all. These are discussed in-depth below.

##### (i) Institutional laws, constitutions, and national policies:

Respondents discussed how health equity was put into practice for the entire population through existing laws, constitutions, and national policies that ensure health for the *entire population*. For example, one respondent highlighted that “[h]ealthcare is a constitutional right in our country. As such, all health programs take into account and ensure that access to care is available to the entire population regardless of age, location and social groups. No marginalization is allowed”. Health policies, plans, and strategies were also frequently mentioned as having health equity as a principle, objective, or as “considering” health equity. Many policymakers described a common sentiment that health equity is a “value, a guiding principle of [country] health reference frameworks”. Health equity was discussed as an important “part of the strategic plan of the health sector”. This became actionable “[i]n all design work undertaken in the area of development strategy” as “the principle of equity is always considered”. Overall, health equity seemed to be integrated “... in the formalization [of policies] at least”.

##### (ii) Ensuring access to health services for all:

Some respondents discussed considering health equity by providing access to health services for all. For example, one respondent expressed the consideration of the larger population (e.g., “everyone having equal chances of opportunities to qualify”—with what an individual would be qualifying for being left unspecified). Another respondent shared that “all populations are covered by health districts, district hospitals, integrated health centers and all benefit from the same technical platform”. Although most initiatives discussed were in terms of specific and targeted population groups, some respondents stated that all services should be or are available for *all*.

#### 3. Through procedural justice.

In addition to taking health equity into account when designing strategies, a few respondents shared information on the direct inclusion of the population in decision-making processes, which aligns with procedural justice. One respondent shared that there is often “a wider stakeholder consultation for every [decision-making] process with the deliberate intention to ensure that the interests of varied population groups including the vulnerable groups are critically considered”. When developing policies, a respondent shared how they have been inclusive of “all categories of people.” An example of how policies can be inclusive of everyone is demonstrated through an attempt to involve different groups to generate evidence to guide policies. However, notably, few respondents described direct inclusion in decision-making or research, as action on health equity.

Although not linked to seeking health equity in practice, respondents shared that there are competing agendas between donors and recipient countries, which is also a procedural justice issue. We discuss this below and note that it is a challenge for evidence-based or -informed decision-making.

#### 4. Operationalizing population health data.

Some respondents alluded to using population health data, in some form, to act on health inequity. Although data sources were not specified, utilizing data was meant to: (i) guide policy and planning, often by aiding in the selection of priority groups or areas for intervention, and (ii) aid in monitoring and evaluation.

Occasionally, “research projects” aligned with the above objectives were mentioned, where data analysis was implied, but without explicitly stating how the data or information is used. For example, one respondent indicated

*At the central level, when a health document is elaborated in a systematic way, we analyze which groups, which regions, which health districts may be disadvantaged, geographically, economically, financially or with respect to a given characteristic, and so we try to address this in the planning. But the problems of inequity are not always solved.*

Similarly, another respondent stated that “[in] the different plans of the Ministry of Health, disadvantaged groups in health are identified, the causes of inequities are identified and solutions to resolve them are addressed.” Therefore, the use of information was implied in the prioritization of target groups in planning.

Respondents also indicated that data was used in monitoring and evaluation. Respondents listed processes or mechanisms informed by data, such as “monitoring, evaluations, assessments, annual reviews, regular follow-up of performance indicators, acts of accountability”. However, only one respondent mentioned interventions: “looking at the equity impact of including interventions in health benefit packages”. Respondents did not elaborate on the disaggregation or “categorizations” of population data explicitly, as the discussion of data was limited. However, respondents alluded to common priority groups throughout their answers, such as women and children and those with a lower socioeconomic status.

When respondents spoke of prioritizing specific groups, “coverage” indicators (e.g., coverage of specific health services, which we assume to be related to UHC indicators) were most commonly described. This is opposed to other types of indicators (e.g., outcome/impact indicators). For example, respondents noted that “Equity in program implementation and resource allocation means ensuring adequate coverage of marginalized groups, whether geographic, socio-cultural or income-related”, “It is part of the strategic plan of the health sector; special support (financial, technical) is provided to areas with low coverage of services”, and “Maternal and child health- What are the coverage indicators for prevention interventions, e.g., what are the utilization indicators.”

### Challenges to incorporating health equity in policy and practice

Despite the foci of health equity efforts described above, respondents also noted challenges for further incorporating health equity. These challenges were related to four key domains: (i) understanding health equity, (ii) governance, (iii) resources, and (iv) lack of data. Notably, respondents’ perceived challenges for materializing health equity in practice were mostly within the second and third domains. Sample excerpts from respondents are noted in Table 1.

**Table 1:** Key challenges with sample quotes from respondents.

Key challenges	Sample quotes
**Understanding health equity**	“Lack of understanding of health equity” “Lack of framework for a dialogue on equity issues” “Identifying who should benefit” “It is the difficulty of identifying sometimes the people who should benefit from aid.” “Contextual factors influencing vulnerability not accounted for in health plans”
**Governance**	“weak governance and leadership” “Our mission is to produce factual bases to inform decision makers in their decision making. Unfortunately most of the time decisions are made for political propaganda, than on an objective basis” “Lack of evidence-based decision making” “Transitioning leadership” “decisions are made at several levels and we need to ensure that technical infor/advice is taken into account by the hierarchy without hindrance” “Donors and partners do not align funds with government priorities, rather, they follow their mandates and values.” “Insecurity” “Terrorism, conflicts, political unrest” “Conflicts and political unrest etc.”
**Resources**	“Lack of resources” “Resource limitations” “Under-resourced” “The availability of budgetary space to support the demand for care” “Inadequate finance” “the lack of financial resources to cover all need” “lack of efficient resource allocation in the sector” “the fight against poverty”, “poverty” “Lack of national health insurance”
**Lack of data**	“we can have indicators but they are not sensitive to highlight certain inequities” “weak data collection” “Time for analysis”

#### 1. Understanding health equity.

Many respondents pointed to challenges with understanding health equity as a limiting factor for incorporating health equity in policy and practice. This was expressed in terms of a general “lack of understanding of health equity” (as a concept), “lack of framework for a dialogue on equity issues”, difficulty in “identifying who should benefit” (i.e., recipients of efforts), and inability to understand individual vulnerability (e.g., “Contextual factors influencing vulnerability not accounted for in health plans”). Evidently, unclarity about what “health equity” means and lacking a basis for discussing this poses an obstacle to action.

#### 2. Governance.

##### (i) Lack of policy, dialogue, and implementation:

Most respondents reported governance-related challenges in materializing health equity in policy and practice, pointing to a range of governance issues within health policy planning and development to implementation.

First, in terms of policy planning and development, there was a lack of “evidence-based decision making” and “lack of framework for a dialogue on equity issues”, and ergo a “lack of common understanding of the [relevant] policy”. The responses alluded to the overall lack of clarity and dialogue around health equity at the policy level. Notably, there was a challenge of “identifying who should benefit” from policy action on health equity, which is a crucial challenge given that many respondents stated that health equity is incorporated in their work through precisely this process — prioritizing certain population groups. Interestingly, one respondent alluded to the lack of flexibility in plans to account for changing dynamics: “the dynamics of contextual factors that can influence vulnerability (plans are not always dynamic enough to take this into account)”.

And second, in terms of policy implementation, responses highlighted the misalignment across levels of governance (e.g., “State policy misaligned for implementation” presumably referring to misalignment between national policy and local level implementation). The need for improved governance mechanisms across different governance levels was suggested: “[D]ecisions are made at several levels and we need to ensure that technical info/advice is taken into account by the hierarchy without hindrance”. Similarly, a “lack of implementation frameworks” for the relevant policy was a challenge, whereby one respondent highlighted a “lack of consideration of specificities in the development and implementation of strategies and its integration at all levels”.

##### (ii) Priority setting and managing competing agendas:

In the realm of governance, two issues emerged related to priority setting and managing competing agendas within countries: (i) competing agendas between donors and recipient countries, and (ii) competing priorities within the national government’s political agenda.

Alluding to the first challenge, respondents stated “donors and funders who are not aligned with government priorities” are a key challenge and “[d]onors and partners do not align funds with government priorities, rather, they follow their mandates and values.” As such, donors who set the agenda without concern for national policy challenges were seen as a challenge for evidence-based or -informed decision-making.

And second, in terms of competing priorities within individual countries, respondents indicated that general instability, insecurity, terrorism, conflict, and political unrest were prioritized at the government level over health equity. This indicates that government officials were not able to work on progressing national and local health equity when a country’s other basic needs were not only unmet but suffering. For example, respondents said their greatest challenge to health equity action was “insecurity [in the country]”, “terrorism, conflicts, political unrest”, and “conflicts and political unrest etc.”. These challenges allude to how action on health equity is impacted by the general state of the country’s politics and political actor network and relationships.

#### 3. Resources.

Funding and resource constraints were also identified as a challenge for further incorporating health equity in practice. The overall lack of resources was the most common resource challenge specified, with respondents heavily using terms like “lack of resources”, “resource limitations”, “under-resourced”, “inadequate finance”, “the lack of financial resources to cover all need[s]”, and “the availability of budgetary space to support the demand for care”. A couple of respondents highlighted the challenge of widespread poverty (e.g., “the fight against poverty”, “poverty”), which arguably implies a similar difficulty as the above, which is the overall lack of financial resources to address health inequity. One respondent specified the “lack of national health insurance” as a challenge to health equity, which also alludes to a financial burden for patients to access care, in addition to governments having the resources to improve health equity. To a lesser extent, respondents highlighted inefficient resource allocation (e.g., “lack of efficient resource allocation in the sector”) and financing for implementation of policies (e.g., “Also the lack of structural financing of health, so that the gaps are well identified, the activities well planned but the conditions of implementation, notably the financing, are not set in motion to tackle the gap”).

#### 4. Lack of data.

Some respondents raised concerns about data challenges for health equity action. First, respondents noted that data on inequities is lacking. For example, “[a challenge for health equity action is the] availability of data on health equity”, “analysis, data and information on vulnerabilities are either not available or partially available to better address these aspects”, and “we can have indicators but they are not sensitive to highlight certain inequities”. Some respondents specified “weak data collection” and “time for analysis” as challenges, which relate to the overall lack of strong data and information on inequities. One respondent mentioned a funding challenge to prioritize data for health equity: “Funding for my department must be available to enable us to provide timely data that lead to public health decisions”. For governments to make evidence-informed decisions to tackle health inequity, data can allow respondents to identify where equity gaps exist. The lack of data also relates to previous challenges, such as the challenge to know how to identify who to prioritize in policy and how to deal with competing agendas.

## Discussion

This study reveals several key findings for public and global health, particularly when considered in connection with academic literature on justice. First, the survey responses revealed that complexities of equity familiar to ethicists were also relevant to respondents in their approaches to health equity. Second, targeting was the predominant approach to health equity. Third, resource constraints are not the only barrier to health equity. And finally, competing priorities and agendas can conflict with anti-colonial approaches and have implications for health equity. Each of these are discussed below.

### Complexities of equity evident in approaches to health equity

The variety of themes raised in the survey responses illustrate the complexity of health equity. Inequities entail unjust differences, and justice is, of course, a multifaceted concept. To begin with, justice can be applied at several levels of analysis. For example, one can ask about the justice of a *distribution* of health resources, but also whether the *process* that led to that distribution was just. Furthermore, at any level of analysis, distinct perspectives on equity exist. Consider this point in connection with distributive justice. Some claim that a just distribution of resources prioritizes the least well-off, a view known as prioritarianism [18]. Another approach, known as sufficientarianism, insists that distributive justice requires everyone to have an adequate set of resources [19]. These two views of distributive justice need not be regarded as mutually exclusive, and some theories of health equity incorporate both [20]. Joint consideration of prioritarian and sufficientarian approaches is also suggested in some WHO documents on health equity. For example, a 2010 WHO report on addressing inequities emphasizes “the need for universal social protection systems (‎ using targeting only for populations that fall through the cracks)” [21].

The aspects of equity just noted have parallels in the survey responses. Thus, associating equity with interventions that target health resources towards especially vulnerable groups, such as people living with HIV or children of low-income families, can be interpreted as an example of prioritarian thinking. Conversely, linking equity to national efforts to ensure universal access to health care could be seen as an example of a sufficientarian approach. Procedural justice concerns are also apparent in some survey responses, such as those associating equity with stakeholder consultation. Responses about priorities being set in a top-down manner by international donors are also related to procedural justice. Again, the concern is that processes determining health priorities and resource distribution do not adequately involve the impacted populations.

Finally, the views on equity described above entail difficult judgments and decisions. To implement a prioritarian approach, it is necessary to decide who is most vulnerable. If resources are scarce, it may not be possible to both prioritize those in greatest need while ensuring that everyone has access to adequate health care. Thus, it is unsurprising that problems of deciding who should benefit from interventions were mentioned by some respondents as a barrier to equity. One goal of future work should then be to provide a framework to support policymakers in articulating and addressing such challenges.

### Targeting was the predominant approach to health equity

Although respondents did not explicitly use the terminology “targeted approach” to describe how they value and mobilize health equity, interventions for “vulnerable” population groups based on a specific criterion were often equated with health equity itself. Health equity was predominantly operationalized through interventions for those with fewer means (e.g., lower socioeconomic status), specific target populations (e.g., women and children), and those with select conditions (e.g., HIV/AIDS, malaria, tuberculosis). Analysis of responses highlighted that very few characteristics (e.g., age, sex, gender, location) were discussed by respondents when describing a targeted approach, but no attention was paid to sexual orientation, race, or Indigeneity. There was also no discussion of intersectionality. Given that characteristics can interact in unique ways to further perpetuate health inequities, differing needs must be considered [22]. However, it is difficult to determine if this resulted because the questionnaire did not elicit information on intersectionality or because it was not a consideration for the responders. As such, we suggest future research explore policymakers’ views on operationalizing intersectional health equity efforts.

### Resource constraints are not the only barrier to health equity

Many respondents noted that resource constraints are a barrier to health equity. Given that initiatives such as providing perinatal care or ensuring access to adequate health care for all inevitably come with a price tag, these responses are entirely understandable. However, we suggest that it would be a mistake to conclude that promoting health equity is solely a matter of securing funding for needed projects. That is because promoting equity is not only about doing *more*; in many cases, it is also about doing things *differently*, for example, through engaging in more equitable partnerships [23]. This point is especially clear in connection with procedural justice. For example, consider a system in which global health priorities are set by donor organizations without adequate input from target countries, mentioned by several respondents as an obstacle to health equity. Changing such a system may be more a matter of political will to reform organizations than acquiring new infusions of funds. Similarly, a lack of understanding of health equity and how to implement it in practice were also noted as barriers. The survey responses, then, suggest that advancing health equity is not only about finding more resources, although that is often important. Reforming top-down processes through which priorities are set and decisions are made and establishing mutual trust may also be crucial.

### Competing priorities and agendas can conflict with anti-colonial approaches and have implications for health equity

Results demonstrated that external agendas (e.g., disease-focused funding such as those from non-governmental actors) may skew national priorities, which can conflict with anti-colonial approaches and reduce procedural equity. In conducting the analysis, it was apparent that many respondents drew on WHO or other United Nations (UN) discourses when discussing priorities. This included mention of the Sustainable Development Goals (SDGs) (“This will help to solve the problem in the right way and reduce injustices to achieve the SDGs”); focus on UHC which is a major priority for the WHO (e.g., “We are currently in the process of setting up universal health coverage”, “We have developed the Universal Health Coverage (UHC) roadmap and implementing it with the aim of achieving the three principles of UHC, namely, access, quality and affordable cost.”); and through historical statements promoted and regurgitated by the WHO (“Leave no one behind” and “Our goal is health for all”). This seeping of WHO and UN language perhaps points to the influence of these international organizations on countries’ policy priorities, agendas, and policy development. With recent attention paid to decolonizing global health and anti-colonial efforts more generally [24], further research is needed to assess the dynamics between international organizations and governments and the plausible influence of international organizations on national public policymaking. For instance, does earmarked funding provided by international organizations sway national policy agendas and associated actions away from national priorities?

Further, if global agendas distract attention from more pressing national issues making it on the policy agenda, this can have implications for health equity. For instance, although noncommunicable diseases (NCDs) have become the leading cause of morbidity and mortality worldwide, they still receive less political and financial attention from the global health community compared to other conditions, such as HIV/AIDS [25–27] as cited in [28]. The focus on the “big three”—HIV/AIDS, malaria, and tuberculosis—which are also prioritized by many global health organizations perhaps reveals the origins of these priorities. In cases where a government prioritizes improving health equity for populations at risk of NCDs, but donor agendas focus on the big three, national health equity efforts for NCDs may be hindered. On the other hand, increased global attention on UHC could shift the focus of national agendas, potentially advancing health equity in areas where it had not been prioritized previously.

### Limitations

This study has limitations in at least four areas: response rate, missing perspectives, computer translation, and survey method.

First, in terms of the response rate, because the complete participant list from the WHO’s Fifth Health Sector Directors’ Policy and Planning Meeting included participants who were not target participants in the study (e.g., translators, observers, report writer), it is impossible to know what denominator to use to accurately determine the response rate. We expect the true response rate to be higher than the 16.5% response rate calculated. Although there is no standard figure for an average response rate, our response rate is not high enough to eliminate concerns. Evidently, there may be various factors contributing to the response rate beyond the control of the researchers, such as survey invitations being filed in spam folders.

Second, despite the survey not having the intention to be representative or generalizable, there were no responses from certain countries and a higher response rate from other countries. For example, there were six responses from the United Republic of Tanzania. Therefore, it is important for future studies to include missing perspectives to have a better understanding of the views of individuals from other countries as well.

Third, because this study was conducted in three languages, we translated French and Portuguese responses into English using computer software to facilitate analysis. Therefore, it is possible that nuances in the use of certain words or expressions were not captured. We expect this limitation to be immensely minimal, as we do not expect any desired meanings to be misinterpreted.

And lastly, there is a recognition that the survey method may not be the most appropriate approach for collecting lengthy response data. Given how surveys are administered, the survey administrator may not be able to interact with respondents in real time despite the trade-off of potentially reaching more respondents. Unlike research methods like interviews or focus groups, respondents are unable to ask questions to clarify the questions and the survey administrator is unable to request additional information if a response is unclear. Without the opportunity to have a conversation or seek and provide clarifications for either party, there may be the possibility of misinterpretations or incomplete responses. We suggest that future studies employ key informant interviews to obtain a better understanding of responses around health equity.

## Conclusion

This study sheds light on the omnipresent, seemingly simple, yet conceptually vague concept of health equity. This novel work demonstrates that there are ongoing policy and programmatic efforts on health equity in the African Region and the findings can inform governmental efforts globally.

We recommend that researchers focus on improving understandings of health equity among policymakers through knowledge translation and exchange. There is often a wide gap between research and practice, but knowledge translation, exchange, and mobilization efforts can help bridge this gap to support actioning health equity in decision-making processes. For instance, workshops can support policymakers to more fulsomely consider health equity, delivered alongside a framework to guide their decisions.

We also recommend that policymakers and those working within donor organizations focus on reforming processes through which priorities are set and decisions are made to actively center health equity concerns at the community, regional, and national levels. We challenge the assumption that acquiring new infusions of funds is always necessary to advance health equity. Equity can be centered as a guiding principle for procedural justice where funds are limited. We can collectively work towards more just health systems regardless of financial constraints.

We suggest that future studies explore policymakers’ perspectives in more depth by employing different methods, such as key informant interviews, to capture richer insights on health equity, including perspectives on intersectionality. Future research should collect missing perspectives from policymakers in countries who did not participate in this study and expand to other WHO regions.

Given that the ways we conduct our work, identify populations of interest, advocate for more equitable health, and fund global health are inherently political [29], it is critical that we do not perpetuate injustice. It is unjust for external actors to dictate national priorities and policies. Adopting transparent, mutually beneficial, and participatory approaches is essential. Ensuring that diverse perspectives—from different sectors, disciplines, backgrounds, and lived experiences—are heard is crucial. Existing research can support the adoption of a more holistic approach (e.g., [30]), helping us learn from shortcoming to ensure health equity remains a central focus (e.g., [31]). Opportunities should be seized to advance health equity [32].
